# Dermatologic toxicities of chemotherapy: an educational intervention for skin of color women with breast cancer

**DOI:** 10.1097/JW9.0000000000000073

**Published:** 2023-01-30

**Authors:** Maham Ahmad, Sabrina Saeed, Brianna Olamiju, Andrea Silber, Jonathan Leventhal

**Affiliations:** a Department of Dermatology, Yale School of Medicine, New Haven, CT; b Yale Cancer Center, Yale School of Medicine, New Haven, CT

**Keywords:** breast cancer, chemotherapy, patient education, pamphlet, skin of color

## Abstract

Minority patients are more likely to require dose adjustments for chemotherapy, with cultural barriers and access to medical care cited as contributory factors.

**Objective:**

We sought to pilot an educational intervention, in the form of a pamphlet, to evaluate the effectiveness of this tool in teaching skin of color (SoC) patients about potential dermatologic toxicities of chemotherapy that are relevant to their skin type.

**Methods:**

At a chemotherapy infusion center, SoC patients (*n* = 26) who were receiving chemotherapy for breast cancer voluntarily consented to read an educational pamphlet and complete a series of survey questions before and after this educational intervention.

**Results:**

Most participants identified as female (96%), African American/Black (81%), and non-Hispanic (85%); all respondents had obtained at least a high school degree. Survey responses revealed a significant increase in knowledge about the potential dermatologic effects of cancer treatment after this intervention. Notably, 100% of participants either agreed or strongly agreed that they would like to see other doctors use this educational tool as a form of patient education, that they would recommend this pamphlet to other patients who are starting cancer treatment, and that the pamphlet was easy to understand.

**Limitations:**

Limitations of this study include small sample size and single-institution recruitment, which may limit generalizability. Furthermore, this study only included patients who are proficient in English.

**Conclusion:**

This study pilots an effective educational tool that addresses dermatologic toxicities of chemotherapy that are relevant to SoC patients. Further multi-institutional studies with larger sample sizes and translation to other languages can overcome the limitations of this pilot study.

**What is known about this subject in regard to women and their families?**

- After skin cancers, breast cancer is the most common malignancy among women worldwide, and its treatment may result in significant dermatologic toxicities; these toxicities can impact women’s quality of life and ability to remain on cancer treatment.
- Women who are skin of color are more likely to require dose adjustments for chemotherapy and suffer worse breast cancer prognosis.

**What is new from this article as messages for women and their families?**

- This study pilots an effective educational tool that addresses dermatologic toxicities of breast cancer chemotherapy that are relevant to women who have skin of color.

## Introduction

Dermatologic toxicities to cancer therapy are common and can have a significant impact on patients’ quality of life; in severe instances, these toxicities may even affect their ability to remain on cancer treatment.^1–3^ Studies have shown that racial minority patients are more likely to require dose adjustments for chemotherapy and suffer worse breast cancer prognosis compared to White patients after controlling for biological factors; cultural barriers and side effects to chemotherapy are cited as contributory factors.^4^ Skin of color (SoC) patients may have particular dermatologic concerns during the course of cancer treatment such as hyperpigmentation, hair and nail care, alopecia, nail infections, as well as the tendency for keloid formation and scarring. Pamphlets may serve as an educational intervention to address these concerns, promote the accessibility of culturally competent medical care, and improve quality of life for this patient population. Thus, the purpose of this study was to design a pamphlet as an educational tool that intentionally includes information relevant to SoC patients and to prospectively evaluate the effectiveness of this tool using self-report questionnaires.

## Materials and methods

This study was approved by the Institutional Review Board of Yale University. Patients were actively recruited between December 2021 and July of 2022. To be eligible for this study, patients were required to be at least 18 years of age, receiving breast cancer treatment, identify with a race other than White/Caucasian, and able to read English. During their infusion treatment, patients at the Smilow Cancer Center at the Yale New Haven Hospital who met the inclusion criteria were asked to read a brief educational pamphlet during their visit, with a short survey of questions required before and after reading the pamphlet. The pamphlet was adapted from a patient education article originally published by the National Cancer Institute, with modifications to include information specifically relevant to SoC patients.^5^ The following additional information was included in the pamphlet: hair and nail care, keloid formation, hyperpigmentation, and reminder to wear sunscreen despite no history of prior sunburn (Supplementary Pamphlet, http://links.lww.com/IJWD/A16).

The survey asked the following questions before and after reading the pamphlet: (1) on a scale of 1-10, “I feel knowledgeable about the potential skin and nail changes during cancer treatment that are relevant to me,” and (2) a quiz question asking if the majority of skin problems related to cancer treatment resolve once treatment has been completed. Following the reading of the pamphlet, a series of questions elicited patients’ feedback regarding the educational intervention using a Likert scale and a free-response section. Survey responses were analyzed using SPSS (version 28.0.0); differences before and after the educational pamphlet were assessed using paired *t* test for means and paired McNemar’s exact test for proportion of correct responses.

## Results

Of the 32 patients who met the inclusion criteria, 26 patients (81%) agreed to participate in this study. The demographics of participants are summarized in Table 1. The majority of respondents identified as female (96%), African American/Black (81%), and non-Hispanic (85%); all respondents had obtained at least a high school degree (Table 1). Notably, analysis of survey responses before and after the pamphlet intervention revealed a significant increase in knowledge about the potential skin and nail changes during cancer treatment, both in terms of subjective rating and correct response to the quiz question (Table 2). A series of questions eliciting feedback from participants on a 5-point Likert scale found that all participants either agreed (35%) or strongly agreed (65%) that the pamphlet increased their knowledge about potential skin, hair, and nail changes from cancer treatment that are relevant to their skin type. Furthermore, all participants either agreed or strongly agreed that they would like to see other doctors use this educational tool as a form of patient education (agreed 31%, strongly agreed 69%), would recommend this pamphlet to other patients who are starting cancer treatment (agreed 31%, strongly agreed 69%), and that the pamphlet was easy to understand (agreed 35%, strongly agreed 65%). At the end of the survey, patients had the option to provide further feedback in free-response format, the results of which are summarized in Table 3.

**Table 1:** Demographics

Characteristic	Value
Total	*n* = 26
Age, years	
Mean (standard deviation)	54.3 (12.8)
Median (range)	53 (29-83)
Sex, *n* (%)	
Female	25 (96)
Male	1 (4)
Nonbinary	0 (0)
Race, *n* (%)	
African American/Black	21 (81)
East Asian/Pacific Islander	1 (4)
South Asian	1 (4)
Other	3 (12)
Ethnicity, *n* (%)	
Hispanic	4 (15)
Non-Hispanic	22 (85)
Education, *n* (%)	
High School	13 (50)
Associate’s degree	6 (23)
Bachelor’s degree	4 (15)
Master’s degree	3 (12)

**Table 2:** Survey responses before and after pamphlet

Question	Preintervention	Postintervention	*P* value
I feel knowledgeable about the potential skin and nail changes during cancer treatment that are relevant to me.	Mean (standard deviation):	Mean (standard deviation):	
On a scale of 1-10	7.38 (3.01)	8.81 (2.04)	<0.001
The majority of skin problems related to cancer treatment resolve once treatment has been completed	*n* (%)	*n* (%)	
True	13 (50)	23 (88)	0.006
False	2 (8)	2 (8)	
I am not sure	11 (42)	1 (4)	

**Table 3:** Qualitative feedback from participants

This pamphlet was incredibly helpful, even though I have been receiving chemo for a while (including last year), there are still some new things that I learned, like wearing sunscreen even though I have never had a sunburn before
This pamphlet helped because it also explains what can happen in different skin types. Maybe it could explain what types of food is good to eat while having chemo also. Also foods to avoid
This pamphlet helped me out, information I did not even know will happen to my skin, great information
Thanks for including a skin of color picture on the front
Really nice to read at the infusion center, I am just sitting around anyways
Now I know what questions to ask my doctor
I did not know about keloids before
This is my first day of chemo, so I found this very helpful. I would love to keep this pamphlet with me to read at home
This information is on point with what I experienced. I also love that this includes things that are relevant to my skin type
It is perfect, easy to read and understand
The pamphlet is a good source
I learned several helpful new things from this pamphlet, for example, that I should not get manicures (I used to think that doing manicures would help)! I also love the reminder about sunscreen
This pamphlet is good amount of information
Thank you for teaching me about these things, very helpful
Easy to understand. A lot of good information. I knew some of it, but not everything. This was very helpful

## Discussion

Racial and ethnic disparities exist in the prognosis of breast cancer, as studies have shown that race is an independent risk factor for survival.^6^ Studies have also found that Black patients are more likely to require modifications in the administration of chemotherapy for breast cancer compared to White patients, an association attributed to psychosocial issues, side effects of chemotherapy, and progression of disease.^4^ Though there is limited data regarding chemotherapy toxicities in skin of color (SoC) patients in particular, some recent studies have found that unique considerations and tailored patient education for hair care are required for this subset of patients undergoing chemotherapy.^7,8^ To our knowledge, this study is the first to pilot an educational intervention for dermatologic toxicities of chemotherapy that includes information specifically for skin of color patients.

Patient education strategies have been proven to be helpful for addressing knowledge gaps and improving treatment adherence for a variety of diseases.^9,10^ Pamphlets in particular have been shown to increase patients’ satisfaction and promote patient empowerment.^11^ For racial minorities and underserved populations, recent studies have assessed the effectiveness of patient educational materials to improve skin cancer prevention practices in SoC patients.^12,13^

In our study, we found that SoC patients felt significantly more knowledgeable about potential skin, hair, and nail changes that may occur during chemotherapy treatment that are relevant to their skin type after the pamphlet educational intervention. The qualitative responses from these patients further highlight the successful features of this pamphlet. Patients noted that the pamphlet was easy to read and included an appropriate amount of information, supported by the fact that the pamphlet was written at a ninth grade reading level based on the Simple Measure of Gobbledygook readability score. This pamphlet also included a separate section for SoC concerns such as hair and nail care, keloid formation, and wearing sunscreen despite never previously being sunburnt. Furthermore, the pamphlet included a list of questions that the patient may ask their healthcare provider to promote shared decision-making and communication with their providers—an important criterion in the DISCERN instrument that assesses quality of health information for patients.^14^

Limitations of this study include small sample size and single-institution recruitment, which may limit generalizability. Furthermore, this pamphlet was designed for patients who are proficient in English, thereby excluding patients who may benefit from this intervention but read or speak another language. Most participants in this study were female, but males may also develop breast cancer and could benefit from this educational tool. Future multi-institutional studies with larger sample sizes that include males and patients of various minority racial and ethnic backgrounds, as well as translation of this pamphlet to other languages, could further assess strengths and weaknesses of such an educational tool for SoC patients undergoing chemotherapy.

## Conflicts of interest

None.

## Funding

None.

## Study approval

Reviewed and approved by Yale IRB, IRB Protocol #2000027657.

## Author contributions

MA: research design, writing manuscript, patient recruitment, data analysis. SS: writing manuscript, patient recruitment. BO: research design, writing manuscript. AS: writing manuscript, supervision. JL: research design, writing manuscript, patient recruitment, supervision.

## Data availability

Data available upon request.

## Acknowledgments

We would like to thank the nurses at the Smilow Cancer Center at Yale for their assistance with recruitment of patients for this study.

## Supplementary Material



## References

[1] Nikolaou V Voudouri D Tsironis G. Cutaneous toxicities of antineoplastic agents: data from a large cohort of Greek patients. Support Care Cancer 2019;27:4535–42. doi:10.1007/s00520-019-04751-y. 30919155 10.1007/s00520-019-04751-y

[2] Lee J Lim J Park JS. The impact of skin problems on the quality of life in patients treated with anticancer agents: a cross-sectional study. Cancer Res Treat 2018;50:1186–93. doi:10.4143/crt.2017.435. 29237254 10.4143/crt.2017.435 PMC6192901

[3] Sibaud V Lebœuf NR Roche H. Dermatological adverse events with taxane chemotherapy. Eur J Dermatol 2016;26:427–43. doi:10.1684/ejd.2016.2833. 27550571 10.1684/ejd.2016.2833 PMC5526115

[4] Smith K Wray L Klein-Cabral M. Ethnic disparities in adjuvant chemotherapy for breast cancer are not caused by excess toxicity in black patients. Clin Breast Cancer 2005;6:260–6; discussion 267. doi:10.3816/CBC.2005.n.029. 16137438 10.3816/CBC.2005.n.029

[5] Skin and Nail Changes during Cancer Treatment. National cancer institute. 2019 [cited 2022 July 16]. Available from: https://www.cancer.gov/about-cancer/treatment/side-effects/skin-nail-changes

[6] Wieder R Shafiq B Adam N. African American race is an independent risk factor in survival from initially diagnosed localized breast cancer. J Cancer 2016;7:1587–98. doi:10.7150/jca.16012. 27698895 10.7150/jca.16012 PMC5039379

[7] Araoye EF Stearns V Aguh C. Considerations for the use of scalp cooling devices in black patients. J Clin Oncol 2020;38:3575–6. doi:10.1200/JCO.20.02130. 32897824 10.1200/JCO.20.02130 PMC7571797

[8] Hrin ML McMichael AJ. Chemotherapy-induced alopecia in African American women: a literature review demonstrates a knowledge gap. J Am Acad Dermatol 2022;86:1434–5. doi:10.1016/j.jaad.2021.06.858. 34197873 10.1016/j.jaad.2021.06.858

[9] Prussick L Tonelli S Gottlieb AB. Improving health literacy and treatment understanding of hidradenitis suppurativa using group educational interventions. J Dermatolog Treat 2019;30:708–13. doi:10.1080/09546634.2018.1562536. 30582391 10.1080/09546634.2018.1562536

[10] Castillo Valladares HB Lee AK Cheraghlou S Zhou A Ramachandran S. A pilot study examining skin cancer education in an underserved population at a free skin cancer screening. Int J Womens Dermatol 2020;7:184–6. doi: 10.1016/j.ijwd.2020.06.007. PMID: 33937488; PMCID: PMC8072505. 33937488 10.1016/j.ijwd.2020.06.007 PMC8072505

[11] Kenny T Wilson RG Purves IN. A PIL for every ill? Patient information leaflets (PILs): a review of past, present and future use. Fam Pract 1998;15:471–9. doi:10.1093/fampra/15.5.471. 9848435 10.1093/fampra/15.5.471

[12] Chao LX Patterson SSL Rademaker AW Liu D Kundu RV. Melanoma perception in people of color: a targeted educational intervention. Am J Clin Dermatol 2017;18:419–27. doi:10.1007/s40257-016-0244-y. 28035649 10.1007/s40257-016-0244-y

[13] Tsai S Frank SH Bordeaux JS. Improving sun-protective behaviors and self-skin examinations among african americans: a randomized controlled trial. Dermatol Surg 2018;44:512–8. doi:10.1097/DSS.0000000000001366. 29016548 10.1097/DSS.0000000000001366

[14] Charnock D Shepperd S Needham G Gann R. DISCERN: an instrument for judging the quality of written consumer health information on treatment choices. J Epidemiol Community Health 1999;53:105–11. doi:10.1136/jech.53.2.105. 10396471 10.1136/jech.53.2.105 PMC1756830

