# Supplementary material for: Dermatologic toxicities of chemotherapy: an educational intervention for skin of color women with breast cancer
Source: Int J Womens Dermatol. 2023 Jan 30;9(1):e073. doi: 10.1097/JW9.0000000000000073 (PMC9886509; doi:10.1097/JW9.0000000000000073)
Supplement: Supplementary file 1 [file jw9-9-e073-s001.pdf]

Cancer treatments may cause hair, skin, and nail changes. Talk with your health care team to learn what side effects your treatment may cause.

Sometimes **radiation therapy** can cause the skin on the part of your body receiving radiation to become dry and peel, itch, and turn red or darker. Your skin may look sunburned or become swollen or puffy. You may develop sores that become painful, wet, and infected.

Some types of **chemotherapy** can cause your skin to become dry, itchy, red or darker, or peel. You may develop a rash or sunburn easily; this is called photosensitivity. Some people also have skin pigmentation changes. Your nails may be dark and cracked, your cuticles may hurt, and your nail plate may lift upward. You may develop full hair loss on certain chemotherapy treatments. For the majority of patients, hair will regrow after completion of treatment. Topical minoxidil (Rogaine) after completion of therapy may increase the rate of hair regrowth. However, up to 1/3 of patients may experience permanent hair changes (hair loss, change in texture, change in color).

Some types of **targeted therapy** may cause dry skin, a rash, or nail problems. If you develop a rash, it is important to talk with your doctor before stopping targeted therapy.

<u>It is important to know that the majority of skin problems resolve once treatment is completed.</u>

Ask Your Health Care Team about Skin and Nail Changes

- What skin and nail changes might I have, based on the cancer treatment I am receiving?
- Which symptoms can be managed at home? Which symptoms need urgent medical care?
- Are there steps I can take to prevent any of these problems?
- When might these problems start? How long might they last?

Skin changes:

- Acne (Acneiform eruptions)
- Blisters
- Burning or skin pain
- Dry skin
- Hand-foot syndrome (palmar-plantar erythrodysesthesia)
- Hyperpigmentation (darker areas of skin, tongue, and joints)
- Hypopigmentation (patches of skin that are lighter)
- Itchy skin (pruritus)
- Peeling, flakey, or crusty skin
- Photosensitivity (sunburn easily)
- Rash
- Red or darkened skin

Hair changes:

- Loss of hair (alopecia)
- Change in hair texture or color

Nail changes:

- Cracked nails
- Cuticles that are swollen and/or painful
- Nail infections (acute paronychia)
- Lifting of nail plate off nail bed (onycholysis)
- Hyperpigmented or yellow nails

Ways to Prevent or Manage Mild Skin and Nail Changes during Cancer Treatment

Depending upon the treatment you are receiving, your health care team may advise you to take these steps:

- **Prevent infection**: Radiation therapy can cause skin in the treatment area to peel, become painful, and wet. Try to keep the area clean and dry so it does not become infected. Patients on chemotherapy treatment should be cautious with use of mascara and false eyelashes as they can increase risk of infection to the sensitive eye area.

Your oncologist at Smilow can refer you to a specialized dermatologist who takes care of patients that develop moderate to severe rashes from their cancer treatment.

Skin of Color

- Sun protection is important during your time on chemotherapy, even if you have never developed a sunburn before, as these medicines can increase sensitivity to the sun in all skin types.
- Patients should consider avoiding chemical relaxers and hair dye with ammonia during chemotherapy treatment since they can accelerate hair breakage.
- Patients with skin of color may be prone to post-inflammatory hyperpigmentation, or dark spots remaining on the skin after a rash. Regular use of sunscreen will prevent hyperpigmented areas from becoming darker. Occasionally hypopigmentation, or spots lighter than your baseline skin color may occur. It may take months for skin complexion changes (hyper- and hypo-pigmentation) to resolve.
- Patients with skin of color may also be prone to keloid formation at their chemotherapy port site. To prevent keloids, patients should keep the site clean, dry, and protected with Vaseline and a nonstick bandage whenever possible.

"Skin and Nail Changes During Cancer Treatment" has been adapted and was originally published by the National Cancer Institute."

- **Use only gentle skin products**. Use mild soaps that are gentle on your skin. If you are receiving radiation therapy, ask about skin products, such as powder that can be used to protect the area after treatment.
- **Protect your skin**: Use sunscreen and sun-protective lip balm. Wear loose-fitting clothing and a hat with a wide brim when outdoors to prevent sunburn.
- **Prevent or treat dry, itchy skin**. Avoid products that list alcohol or fragrance as an ingredient, since it can dry or irritate your skin. Take short showers or baths in lukewarm, not hot, water. Apply recommended creams or lotions after drying off from a shower, but while your skin is still damp.
- **Prevent or treat minor nail problems**. Keep your nails clean and cut short to avoid accidentally tearing them, though avoid cutting the cuticles. Protect your hands and nails by wearing gloves when you wash the dishes, or clean the house. Avoid getting frequent manicures and pedicures.
- **Learn about treatments for irritating or painful skin rashes**: Sometimes severe skin problems require medical treatment. Your rash may be treated with a medicated cream (topical corticosteroids) or with medicine that you take as a pill (oral corticosteroids or antibiotics).

Skin, Hair, and Nail Changes During Cancer Treatment

Including for Skin of Color

[Insert skin of color photograph here]

Supplemental Digital Content 1. Pamphlet
